# Heterogeneity of Human Breast Stem and Progenitor Cells as Revealed by Transcriptional Profiling

**DOI:** 10.1016/j.stemcr.2018.03.001

**Published:** 2018-03-29

**Authors:** Justin A. Colacino, Ebrahim Azizi, Michael D. Brooks, Ramdane Harouaka, Shamileh Fouladdel, Sean P. McDermott, Michael Lee, David Hill, Julie Madden, Julie Boerner, Michele L. Cote, Maureen A. Sartor, Laura S. Rozek, Max S. Wicha

**Affiliations:** 1 Department of Environmental Health Sciences, University of Michigan School of Public Health, Ann Arbor, MI, USA; 2 Department of Nutritional Sciences, University of Michigan School of Public Health, Ann Arbor, MI, USA; 3 Comprehensive Cancer Center, University of Michigan, Ann Arbor, MI, USA; 4 Department of Internal Medicine, University of Michigan Medical School, Ann Arbor, MI, USA; 5 Department of Oncology, Wayne State University School of Medicine, Detroit, MI, USA; 6 Population Sciences and Health Disparities Program, Karmanos Cancer Institute, Detroit, MI, USA; 7 Department of Computational Medicine and Bioinformatics, University of Michigan Medical School, Ann Arbor, MI, USA

**Keywords:** breast stem cell, single-cell RNA, epithelial, mesenchymal, hybrid, RNA-seq

## Abstract

During development, the mammary gland undergoes extensive remodeling driven by stem cells. Breast cancers are also hierarchically organized and driven by cancer stem cells characterized by CD44^+^CD24^low/−^ or aldehyde dehydrogenase (ALDH) expression. These markers identify mesenchymal and epithelial populations both capable of tumor initiation. Less is known about these populations in non-cancerous mammary glands. From RNA sequencing, ALDH^+^ and ALDH^−^CD44^+^CD24^−^ human mammary cells have epithelial-like and mesenchymal-like characteristics, respectively, with some co-expressing ALDH^+^ and CD44^+^CD24^−^ by flow cytometry. At the single-cell level, these cells have the greatest mammosphere-forming capacity and express high levels of stemness and epithelial-to-mesenchymal transition-associated genes including *ID1*, *SOX2*, *TWIST1*, and *ZEB2*. We further identify single ALDH^+^ cells with a hybrid epithelial/mesenchymal phenotype that express genes associated with aggressive triple-negative breast cancers. These results highlight single-cell analyses to characterize tissue heterogeneity, even in marker-enriched populations, and identify genes and pathways that define this heterogeneity.

## Highlights

• Isolation and RNA-seq of ALDH^+^ and CD44^+^CD24^−^ breast cells
• Unlike in cancer, there is substantial overlap in ALDH^+^ and CD44^+^CD24^−^ populations
• Single-cell analysis of ALDH^+^ cells identifies unexpected subpopulation structure
• Hybrid epithelial/mesenchymal ALDH^+^ cells have a cancer-like expression signature

## Teaser

In this article, Colacino and colleagues use flow-cytometry-sorted populations and single-cell analyses to investigate human mammary stem cells. They discover unexpected phenotypic and functional heterogeneity at the single-cell level, including a subpopulation of ALDH^+^ stem cells with a hybrid epithelial/mesenchymal phenotype and triple-negative breast cancer-like gene expression pattern.

## Introduction

*In utero*, throughout puberty, and during pregnancy, the human mammary gland undergoes extensive expansion and remodeling, driven by populations of stem and progenitor cells (Visvader and Stingl, 2014). The mammary epithelium consists of two major cell lineages, luminal and myoepithelial. Lineage tracing shows the mammary differentiation hierarchy consists of bipotent stem cells that give rise to both luminal and myoepithelial cells (Rios et al., 2014), as well as long-lived unipotent progenitor cells that drive mammary gland development and homeostasis (Van Keymeulen et al., 2011). Breast cancers also display a differentiation hierarchy and are driven by a stem -like population (Al-Hajj et al., 2003). The long-lived nature and proliferative capacity of bipotent stem cells or lineage-committed progenitor cells make these good candidates to be breast cancer cells of origin. Molecular analysis of breast cancers led to identification of subtypes with distinct gene expression profiles and clinical behaviors. This led to the hypothesis that different breast tumor subtypes arise from cells in the normal breast hierarchy (Skibinski and Kuperwasser, 2015). Despite substantial advances in the field, it remains unclear whether the differentiation hierarchy in breast cancers reflects that in the normal breast.

We have reported that aldehyde dehydrogenase activity (termed ALDH^+^) and CD44^+^CD24^−^ mark two largely non-overlapping populations of cancer stem cells, which have epithelial-like and mesenchymal-like phenotypes, respectively (Liu et al., 2013). These cells are plastic and can interconvert, in a process driven by the tumor microenvironment. This plasticity may play an important role in the successful execution of metastasis (Liu et al., 2013). The existence of these two stem cell populations mirrors that of the long-lived unipotent progenitor populations in the normal breast. The markers ALDH^+^ (Ginestier et al., 2007) and CD44^+^CD24^−^ (Choudhury et al., 2013, Shipitsin et al., 2007) have also proved useful in the isolation and functional characterization of normal human breast stem cells. Other markers, such as CD49f^hi^EpCAM^−/lo^ (Stingl et al., 2001, Visvader, 2009), have also been reported to enrich for normal human breast stem cells. The importance of the ALDH^+^ and CD44^+^CD24^−^ cell populations in breast cancer, and the comparative lack of knowledge about cells that express either or both markers in the normal breast, highlight the importance of further characterizing these populations.

The goal of this study was to understand the biology of the ALDH^+^ and CD44^+^CD24^−^ cells in the human breast, using flow-cytometry-based sorting of cells isolated from reduction mammoplasty tissues paired with functional *ex vivo* analyses, RNA sequencing (RNA-seq), and single-cell RNA profiling. Unlike in breast cancers, we identified a significant overlap between the ALDH^+^ and CD44^+^CD24^−^ populations, with substantial interindividual variation in the degree of overlap. While ALDH^+^ cells and ALDH^−^CD44^+^CD24^−^ (hereafter referred to as CD44^+^) cells generally represent epithelial-like and mesenchymal-like populations, there are similarities in the biological pathways activated in both populations when compared with differentiated ALDH^−^CD44^−^CD24^+^ (hereafter referred to as CD24^+^) cells. The cells that express both ALDH^+^ and CD44^+^CD24^−^ have the greatest mammosphere formation potential, and express higher levels of stemness and epithelial-to-mesenchymal transition (EMT)-related genes. By conducting an unbiased analysis of single cells, we identified substantial cellular heterogeneity within the ALDH^+^ and CD44^+^/CD24^−^ populations. In addition, we demonstrate the existence of a subpopulation of ALDH^+^ cells that simultaneously express both epithelial and mesenchymal markers. Expression of these markers is associated with poor outcome in triple-negative breast cancer (TNBC) patients.

## Results

### Isolation and Characterization of Human Mammary Cell Populations

**Figure 1:**
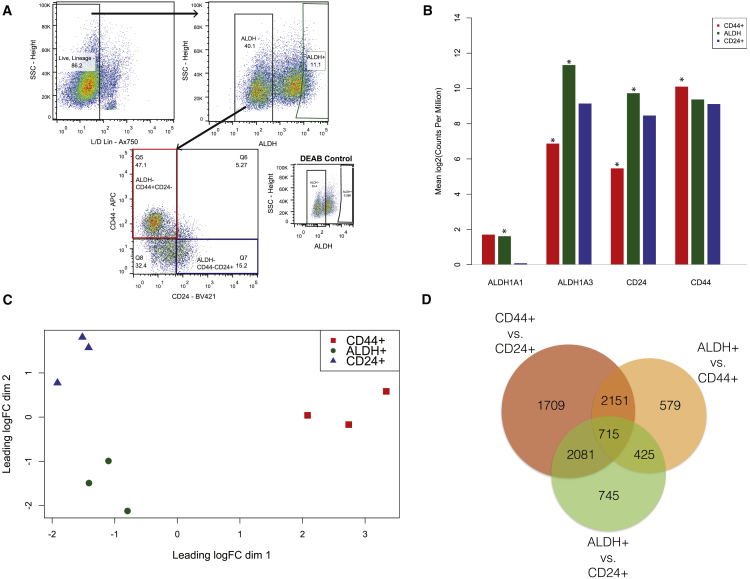
Purification and Transcriptomic Profiling of ALDH^+^, ALDH^−^CD44^+^CD24^−^, and ALDH^−^CD44^−^CD24^+^ Human Breast Cells (A) A representative FACS isolation diagram of the three populations of cells isolated from reduction mammoplasties. ALDH^+^ gating was based on the DEAB negative control. ALDH^−^CD44^+^CD24^−^ will be hereafter referred to as CD44^+^ and ALDH^−^CD44^−^CD24^+^ as CD24^+^. (B) RNA expression, from RNA-seq analysis of FACS-purified cells from three donors, of genes associated with the sorting markers. ^∗^False discovery rate (FDR) p < 0.05. (C) Multidimensional scaling plot based on the 500 most variably expressed genes. (D) Overlap in differentially expressed (FDR p < 0.05) genes between the three populations.

To follow up on our findings of epithelial-like and mesenchymal-like breast cancer stem cells (Liu et al., 2013), we isolated three cellular populations from reduction mammoplasty samples (n = 3 independent biological replicates) by flow cytometry: ALDH^+^, CD44^+^, and CD24^+^ (Figure 1A). Through RNA-seq, we confirmed that expression of *ALDH1A1*, *ALDH1A3*, *CD44*, and *CD24* matched the protein markers used for sorting (Figure 1B). Multidimensional scaling identified that the samples cluster on the first two dimensions of the leading log fold change, with ALDH^+^ and the CD24^+^ cells grouping together on the first dimension but separating on the second (Figure 1C). Differential expression analysis identified broad gene expression differences between the populations (Figure 1D).

### The ALDH^+^ Breast Cell Gene Expression Signature

**Figure 2:**
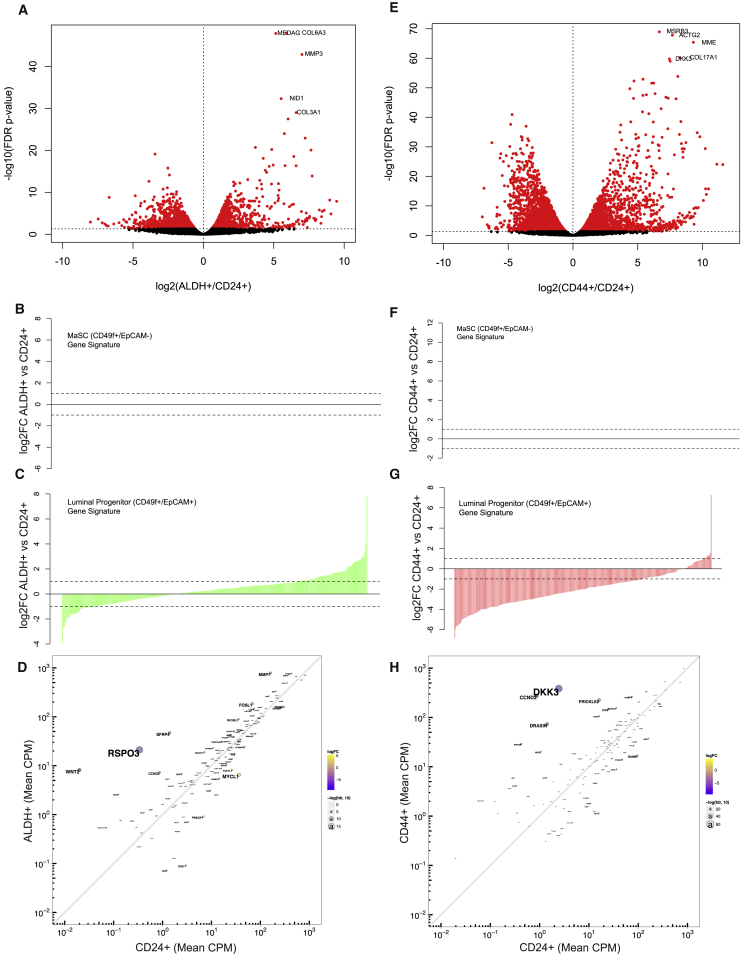
Comparison of Gene Expression Signatures between ALDH^+^ and CD44^+^ with Non-stem Cell-Enriched CD24^+^ Cells (A) FDR volcano plot comparing the change in gene expression between ALDH^+^ and CD24^+^ cells from three donors, with the names of the top five most statistically different genes labeled. (B and C) Comparison of the log_2_ fold-change differences between ALDH^+^ and CD24^+^ and the mammary stem cell and luminal progenitor cell gene expression signature, respectively, reported in Lim et al. (2009). (D) Enrichment of Wnt signaling genes in ALDH^+^ relative to CD24^+^ cells. (E) FDR volcano plot comparing differences in gene expression between CD44^+^ and CD24^+^ cells. (F and G) Comparison of the log_2_ fold-change differences between CD44^+^ and CD24^+^ (F) and the mammary stem cell and luminal progenitor cell gene expression signature (G), respectively. (H) Enrichment of Wnt signaling-related genes in CD44^+^ relative to CD24^+^ cells.

We have previously shown that ALDH^+^ normal breast and breast cancer cells are enriched for stem-like cells (Ginestier et al., 2007). To quantify expression patterns specific to ALDH^+^ cells, we compared expression of ALDH^+^ cells with that of CD24^+^ cells, which do not express the canonical breast stem cell markers ALDH or CD44^+^/CD24^−^. In ALDH^+^ cells, 2,244 genes were upregulated and 1,730 downregulated (Figure 2A and Table S1). The top three most overexpressed genes, by magnitude, were *WNT2* (fold change = 705.3), insulin-like growth factor 1 (*IGF1*; fold change = 532.3), and the notch ligand *DLL1* (fold change = 502.5). We next compared the ALDH^+^ cell expression signature with previously reported gene expression signatures of human mammary stem (CD49f^+^/EpCAM^−^) and luminal progenitor (CD49f^+^/EpCAM^+^) cells (Lim et al., 2009). We did not observe strong enrichment for either the mammary stem or luminal progenitor gene signature in ALDH^+^ cells (Figures 2B and 2C). Analyzing relative expression of WNT pathway genes showed that, in addition to *WNT2*, ALDH^+^ cells also overexpress *RSPO3*, *SFRP4*, *MMP7*, and *FOSL1* (Figure 2D). KEGG pathway analyses identified that ALDH^+^ cells differentially expressed genes involved in ribosome (false discovery rate [FDR] = 3.1E−16), oxidative phosphorylation (FDR = 2.6E−14), and the proteasome (FDR = 7.2E−14) (Figures S1A–S1C). In each of these three pathways, all of the differentially expressed genes were upregulated in the ALDH^+^ cells. Genes differentially expressed in ALDH^+^ cells were also enriched in pathways related to focal adhesion (p = 1.9E−4) and extracellular matrix (ECM)-receptor interactions (p = 9.8E−7) (Table S2).

### The ALDH^−^CD44^+^CD24^−^ Gene Expression Signature

We further compared the gene expression profile of CD44^+^ cells with that of CD24^+^ cells and identified 3,361 genes upregulated and 3,295 genes downregulated (Figure 2E and Table S3). The most overexpressed genes by magnitude in the CD44^+^ cells were insulin-like growth factor binding protein 1 (*IGFBP1*; fold change = 3,040.3), the glycosyltransferase *ST8SIA2* (fold change = 2,210.3), phospholipase D family member 5 (*PLD5*, fold change = 1,418.4), the chaperone protein *SCG5* (fold change = 1,269.5), and the cytoskeletal protein *MYOT* (fold change = 1,209.3). These genes had no detectable expression in CD24^+^ cells (Table S3). The expression pattern of Wnt pathway members in CD44^+^ cells was different than for ALDH^+^ cells, with CD44^+^ cells expressing *DKK3*, *CCND2*, *PRICKLE2*, and *DRAXIN* (Figure 2H). CD44^+^ cells had enrichment for the previously reported mammary stem (CD49f^+^/EpCAM^−^) gene signature (Figure 2F) and negative enrichment for the luminal progenitor signature (Figure 2G). CD44^+^ cells overexpress genes associated with the proteasome (FDR = 3.8E−8) and ECM-receptor interactions (FDR = 5E−6), and differentially express genes associated with focal adhesion (FDR = 1.6E−6) (Figures S2A–S2C). Furthermore, CD44^+^ cells differentially expressed genes involved in phosphatidylinositol 3-kinase-AKT signaling (FDR = 7.0E−5), including *CCDN2*, *PIK3CG*, *FGF1*, and *NGF* (Figure S2D).

### CD44^+^ Cells Express Mesenchymal Markers, whereas ALDH^+^ Cells Express Both Epithelial and Mesenchymal Markers

**Figure 3:**
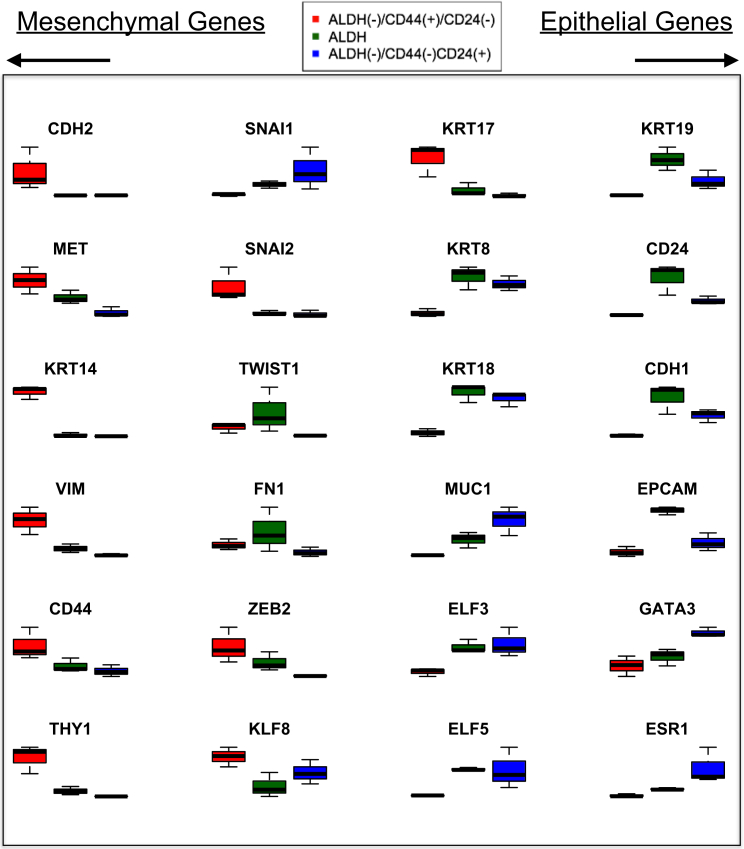
Relative Expression Levels of Mesenchymal Phenotype- and Epithelial Phenotype-Associated Genes in ALDH^+^, CD44^+^, and CD24^+^ Cells from Three Donors

We previously showed that ALDH^+^ cells cultured as mammospheres for 24 hr had a global gene expression pattern reflecting an epithelial phenotype relative to CD44^+^ cells, which have a mesenchymal phenotype (Colacino et al., 2016). To further explore the relative phenotypes of the ALDH^+^, CD44^+^, and CD24^+^ cells, we compared the expression of genes associated with an epithelial or mesenchymal phenotype (Figure 3). In general, CD44^+^ cells were enriched for expression of mesenchymal markers, including *CDH2*, *KRT17*, *ZEB2*, *KLF8*, *CD44*, *KRT14*, and *VIM*. ALDH^+^ cells expressed the highest levels of epithelial markers, including *KRT19*, *CD24*, *CDH1*, *EpCAM*, and *KR18*. ALDH^+^ cells also, however, expressed intermediate or highest levels of several mesenchymal markers, including the EMT transcription factors *SNAI1*, *TWIST1*, and *ZEB2*. These results suggest that ALDH^+^ cells are simultaneously expressing both epithelial and mesenchymal markers or that there are epithelial-like and mesenchymal-like subpopulations within the ALDH^+^ cell fraction.

### ALDH^+^ Cell Populations Are Highly Variable among Individuals

**Figure 4:**
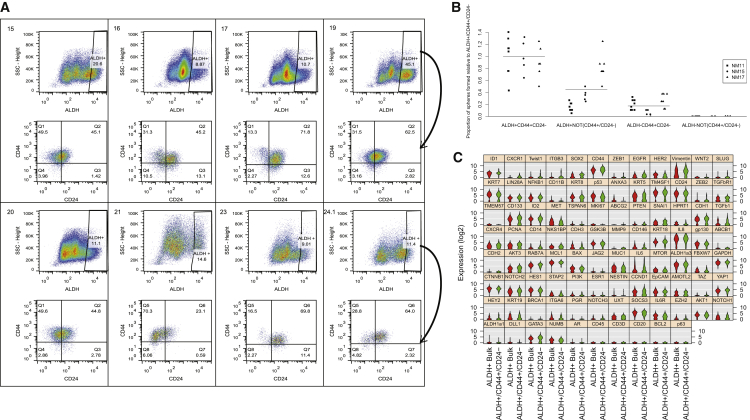
Quantitation and Profiling of Breast Cells that Express Both Stem Cell Markers ALDH^+^ and CD44^+^CD24^−^ (A) Quantitation, by flow cytometry, of the ALDH^+^ cell population in mammary tissues (n = 8). ALDH^+^ cells were further analyzed for CD44 and CD24 expression (arrow), with the top left quadrant in each bottom panel representing ALDH^+^CD44^+^CD24^−^ cells. (B) Mammosphere formation rates of cells expressing different combinations of ALDH and CD44/CD24 (cells sorted from n = 3 individuals: termed NM11, NM15, NM17, at least five technical replicates run per condition with results from all replicates shown), with formation rates presented relative to ALDH^+^CD44^+^CD24^−^ cells. (C) Single-cell gene expression profiling of ALDH^+^CD44^+^CD24^−^ and ALDH^+^ bulk (ALDH^+^ cells that do not express CD44^+^CD24^−^) cells. Genes are ordered by statistical significance of difference in expression between the two populations.

To better understand the heterogeneity within ALDH^+^ cells, we conducted flow-cytometry analysis of ALDH activity combined with CD44 and CD24 staining across reduction mammoplasty samples from eight women. We identified significant interindividual variation in the proportion of ALDH^+^ cells (range 8.9%–45%) (Figure 4A). When analyzing the relative proportion of CD44^+^CD24^−^-expressing cells within the ALDH^+^ fraction, we identified further heterogeneity between individuals. The proportion of CD44^+^CD24^−^ cells within the ALDH^+^ fraction ranged from 13.3% to 70.3%. The majority of ALDH^+^ cells tended to also be CD44^+^. To understand the functional differences between these cell populations, we isolated four populations by fluorescence-activated cell sorting (FACS)—ALDH^+^CD44^+^CD24^−^ cells, ALDH^+^ cells that are not CD44^+^CD24^−^, ALDH^−^CD44^+^CD24^−^ cells, and ALDH^−^ cells that are not CD44^+^CD24^−^—and determined their capacity to form mammospheres. ALDH^+^CD44^+^CD24^−^ cells had the highest mammosphere-forming capacity. Both ALDH^+^ cells that are not CD44^+^CD24^−^ and ALDH^−^CD44^+^CD24^−^ also formed mammospheres, but at rates less than ALDH^+^CD44^+^CD24^−^ cells (Figure 4B). ALDH^−^ cells that are not CD44^+^CD24^−^ cells did not form mammospheres. These results suggest that the majority of the cells with mammosphere-forming potential lie in the ALDH^+^CD44^+^CD24^−^ cell fraction.

### Single-Cell RNA Profiling Reveals Further Heterogeneity within the ALDH^+^ Cell Population

To characterize the expression differences that define ALDH^+^CD44^+^CD24^−^ cells and the remainder of ALDH^+^ cells, we isolated these two fractions from three independent donors by FACS, fluorescently labeled each population, and subjected them to both bulk RNA and single-cell RNA expression analysis of a custom 96-gene panel. This panel contains genes previously demonstrated to be expressed in stem cells or in regulating EMT (Holm et al., 2010, Jolly et al., 2015, Liu et al., 2013, Cancer Genome Atlas Network, 2012). Utilizing the RNA-seq data described above, we confirmed that the custom panel effectively discriminated ALDH^+^, CD44^+^, and CD24^+^ cells populations by multidimensional scaling (Figure S3A).

Comparing RNA isolated from bulk ALDH^+^CD44^+^CD24^−^ and ALDH^+^ cells that are not also CD44^+^CD24^−^, we identified that ALDH^+^CD44^+^CD24^−^ cells, on average, expressed lower levels of *BAX*, *CDH3*, *CDH1*, *KRT19*, and *MUC1* (p < 0.10; Figure S3B and Table S4). To characterize the heterogeneity present within these sorted populations, we analyzed expression of the 96-gene panel in single cells isolated by Fluidigm C1 technology. When comparing gene expression profiles at the single-cell level, a different pattern emerged (Figure 4C). At the single-cell level, ALDH^+^CD44^+^CD24^−^ cells overexpress the stem cell genes *ID1* and *SOX2*, the interleukin-8 receptor *CXCR1*, and the EMT-associated transcription factor *TWIST1*. ALDH^+^CD44^+^CD24^−^ cells also significantly overexpressed *EGFR*, *CD44*, and *VIM*, while expressing *HER2* at lower levels.

**Figure 5:**
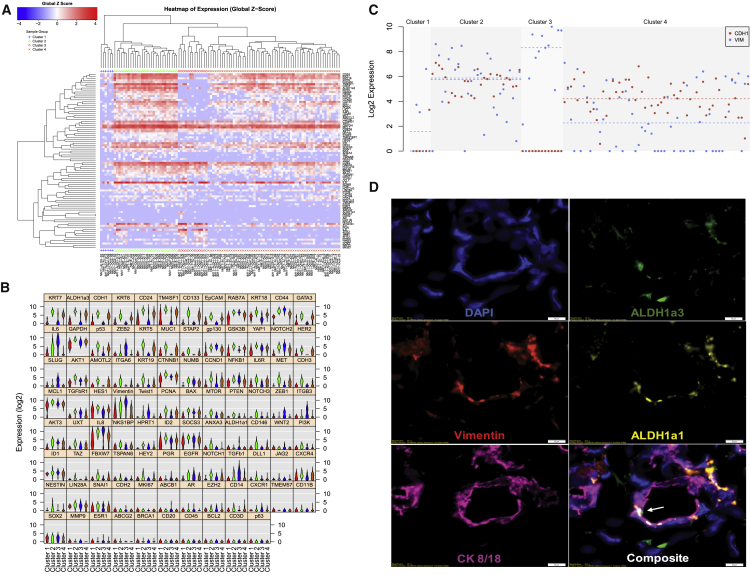
Unbiased Analysis of Single-Cell Gene Expression Data from ALDH^+^ Breast Cells (A) Hierarchical clustering analysis of gene expression measures from a total of 105 ALDH^+^ cells isolated from three independent individuals reveals four expression clusters. (B) Violin plot analysis of the expression of the gene expression panel across the four clusters. (C) Comparison of expression of the epithelial gene *CDH1* (red) and the mesenchymal gene *VIM* (blue) across the four clusters. (D) Immunofluorescence antibody staining of adjacent normal breast tissue for DAPI, ALDH1A1, ALDH1A3, CK8/18, and vimentin. Arrow identifies cell with co-expression of CK8/18, vimentin, and ALDH1A3. Scale bars, 10 μm.

To further explore the heterogeneity of ALDH^+^ cells, we conducted unsupervised hierarchical clustering of gene expression of single ALDH^+^ cells (n = 105 total cells collected from three individuals). We observed four clusters (Figure 5A) with substantial expression differences, with 75 of the genes being significantly different across the groups by ANOVA (p < 0.05; Figure 5C and Table S5). Cluster 1 was characterized, in general, by low overall gene expression. Cluster 4 expressed high levels of epithelial-related genes, including *KRT7*, *CD24*, *EPCAM*, *GATA3*, and *KRT5*. Cluster 3 was characterized by expression of mesenchymal phenotype related genes, including *CD44*, *ZEB2*, *SLUG*, *VIM*, *TWIST1*, and *TGFB1*. Cluster 2 had the highest average expression of *ITGA6*, *CD133*, and *ALDH1A3* and expressed high levels of epithelial genes, such as *KRT7*, *KRT8*, and *CDH1* as well as EMT genes, including *IL6*, *CD44*, *TM4SF1*, and *VIM*, and the mesenchymal stem cell marker *CD146*. Cells in this cluster express canonical markers of breast stemness at the highest levels and present with a hybrid epithelial/mesenchymal phenotype (Figure 5B). Individual cells in cluster 2 simultaneously expressed both the epithelial marker *EPCAM* and the mesenchymal marker *VIM* (Figure 5C). Cluster membership was found to be associated with the patient from whom the cells were isolated (Figure S4), with the majority of cluster 2 cells belonging to patient NM15. This emphasized the variability of these cell populations across individuals. To validate hybrid epithelial/mesenchymal cells, we performed multiparameter immunofluorescence in additional patient-derived breast tissues adjacent to breast cancers. In general, we observed that cells expressing epithelial (KRT8/18) and mesenchymal (vimentin) markers remained distinct (Figure S5). However, we also identified rare cells co-expressing KRT8/18 and vimentin, as well as ALDH1A3 (Figures 5D and S5B), confirming that these markers co-localize *in situ*.

### ALDH1A1-Expressing Cells Have a Mesenchymal-like Phenotype

Cells in cluster 3 expressed the highest levels of *ALDH1A1* and low levels of *ALDH1A3*, while cells in clusters 2 and 4 expressed high levels of *ALDH1A3* but low levels of *ALDH1A1*. This segregated expression of *ALDH1A1* and *ALDH1A3* corroborates reports of differential localization of ALDH1A1 and ALDH1A3 in the normal breast (Honeth et al., 2015). We compared the expression patterns between *ALDH1A1*-expressing cells and the remainder of the ALDH^+^ cells (Figure S6). *ALDH1A1*^+^ cells expressed higher levels of mesenchymal markers, including *TWIST1*, *ZEB2*, *IL6*, *VIM*, and *TGFB1*, and lower levels of epithelial markers *CDH1*, *CD24*, and *EpCAM*. *ALDH1A1*^+^ cells also expressed higher levels of androgen receptor (*AR*) (Table S6). Using immunofluorescence, we identified cells that co-expressed ALDH1A1 and vimentin in the absence of KRT8/18, providing supporting evidence for ALDH1A1^+^ cells with a mesenchymal phenotype (Figure S5C).

### Clinical Relevance of ALDH^+^-Associated Genes in Breast Cancer

**Figure 6:**
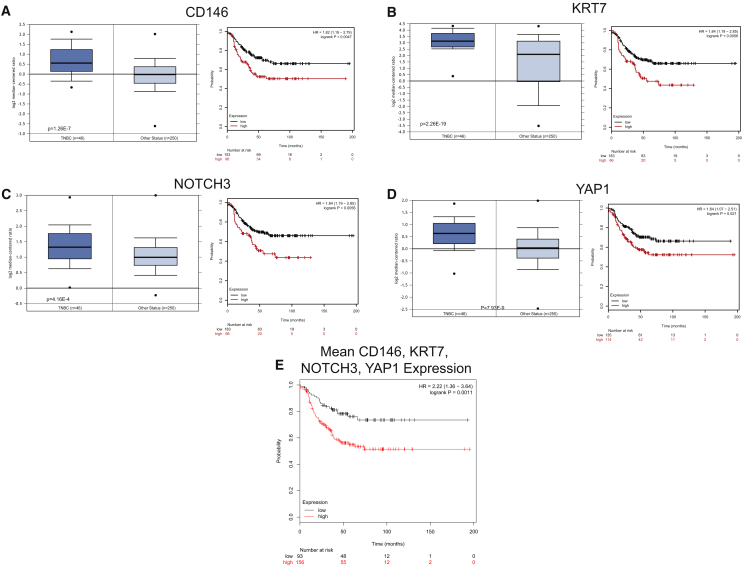
Expression of Cluster 2 Genes in TNBCs and the Relationship of Expression with Survival (A–D) Expression of *CD146* (A), *KRT7* (B), *NOTCH3* (C), and *YAP1* (D) in TNBCs relative to other breast cancers as well as relative survival of patients with TNBCs that express high relative to low amounts of each gene. (E) Relative survival of TNBC patients with tumors that have high relative to low mean *CD146*, *KRT7*, *NOTCH3*, and *YAP1* expression. HR, hazard ratio.

In light of our findings that within ALDH^+^ cells, cells that expressed both mesenchymal and epithelial genes had the greatest mammosphere-forming capacity, we wondered whether the gene signature of cells with these properties had clinical relevance in breast cancer. We identified four genes that were overexpressed in cluster 2 cells—*KRT7*, *NOTCH3*, *CD146*, and *YAP1*—and analyzed publicly available expression data to assess the relevance of these genes in breast cancer (Figures 6A–6D). These genes were overexpressed in TNBCs compared with other breast cancers in the Cancer Genome Atlas. This is consistent with previous reports for enrichment of cancer stem cells in this subtype (Idowu et al., 2012, Yoshioka et al., 2011). Furthermore, overexpression of these four genes was associated with worse survival in TNBC patients, and patients with high mean expression of all four genes had significantly worse survival (Figure 6E, hazard ratio = 2.22). These results suggest that gene expression programs in cluster 2 ALDH^+^ cells are dysregulated in the most aggressive TNBCs.

## Discussion

The results presented here highlight the inter- and intraindividual heterogeneity of the stem/progenitor cell populations of the human mammary gland. By conducting RNA-seq on FACS-isolated ALDH^+^ and CD44^+^ cells, we found significant differences, as well as similarities, between these two populations when compared with differentiated cells. CD44^+^ express high levels of mesenchymal-related markers, while ALDH^+^ cells express high levels of epithelial-associated markers but also express intermediate levels of some mesenchymal-associated markers. We identified a considerable amount of variation in the proportion of ALDH^+^ cells between individuals, and further substantial variation between individuals when ALDH^+^ cells were subsequently analyzed for their expression of CD44 and CD24. Cells that express both ALDH and CD44^+^CD24^−^ have the highest mammosphere-forming potential. At the single-cell level, compared with the rest of ALDH^+^ cells, ALDH^+^CD44^+^CD24^−^ cells overexpress EMT and stemness genes. By unbiased clustering of ALDH^+^ single-cell RNA expression data, we identified individual cells with epithelial-like, mesenchymal-like, or dual epithelial/mesenchymal phenotypes. Within the cells that expressed both epithelial and mesenchymal markers, there was high expression of genes overexpressed in TNBC and an association with poor survival in these patients.

Our previous work showed that breast cancer stem cells express CD44^+^CD24^−^ (Al-Hajj et al., 2003) or ALDH^+^ (Ginestier et al., 2007), and that these two markers isolate distinct populations of mesenchymal-like and epithelial-like cells, respectively (Liu et al., 2013). Despite the lack of overlap between these populations, these cells maintain cellular plasticity, allowing them to interconvert in a process regulated by the tumor microenvironment (Liu et al., 2013). Similarly, ALDH^+^ or CD44^+^ cells isolated from reduction mammoplasty and cultured as mammospheres also have distinct epithelial or mesenchymal phenotypes relative to each other (Colacino et al., 2016). In the current study, we expanded upon these findings using CD24^+^ cells as a differentiated cell anchor to compare the expression signatures of ALDH^+^ and CD44^+^ cells. CD44^+^ cells are enriched for the mammary stem cell gene signature of CD49f^+^/EpCAM^−^ cells (Lim et al., 2009). In contrast, there was no clear enrichment for either the mammary stem cell or the luminal progenitor gene signature in ALDH^+^ cells. Despite large gene expression and phenotypic differences between CD44^+^ and ALDH^+^ cells, when compared with CD24^+^ cells there was substantial overlap in differentially expressed biological pathways, including the proteasome, oxidative phosphorylation, focal adhesion, and ECM-receptor interactions. ALDH^+^ and CD44^+^ cells displayed similar patterns of expression of genes associated with the proteasome and oxidative phosphorylation. Findings of increased proteasome activity in these two populations corroborate previous findings in embryonic stem cells (Jang et al., 2014, Vilchez et al., 2012) and neural progenitor cells (Zhao et al., 2016). Intriguingly, breast cancer stem cells may have lower proteosomal activity (Vlashi et al., 2009, Vlashi et al., 2013), suggesting that downregulation of the proteasome may be a key step in the generation of breast cancer stem cells. Both ALDH^+^ and CD44^+^ cells also expressed higher levels of genes involved in oxidative phosphorylation. While it has been assumed that the stem cell niche is hypoxic and that stem cells preferentially utilize glycolysis for energy production (Shyh-Chang et al., 2013), emerging data also point to a number of proliferative undifferentiated cell populations utilizing oxidative phosphorylation, including osteoblasts (Chen et al., 2008) and preadipocytes (D’Esposito et al., 2016, Tormos et al., 2011). Finally, compared with CD24^+^ cells, both CD44^+^ and ALDH^+^ cells significantly differentially express genes associated with focal adhesion and interactions with the ECM, although the expression patterns between the two stem cell-enriched fractions are very different. ECM-interacting receptors and proteins, including cadherins and integrins, are known regulators of stem cell self-renewal and differentiation (Gattazzo et al., 2014). These interactions may play an important role in the stem cell niche. For example, *ITGB3* was expressed at higher levels in ALDH^+^CD44^+^CD24^−^ cells at the single-cell level, and in CD44^+^, compared with CD24^+^, cells in sorted population analyses, suggesting that this integrin molecule may be associated with CD44 expression. Further exploration of the influence of these pathways in the regulation of breast stem cells will likely provide insights into breast cancer stem cell biology.

We found substantial heterogeneity in proportions of ALDH^+^ stem cells between individuals. Moreover, there was considerable interindividual variation in CD44^+^CD24^−^ expression within the ALDH^+^ population. Our findings corroborate previous work that found interindividual heterogeneity in the ALDH^+^ population in normal breast cells isolated from breast punch biopsies and grown in conditional reprogramming conditions (Nakshatri et al., 2015). It is currently unknown which epidemiologic, clinical, or environmental risk factors contribute to this variation, although this is an area of research interest for us moving forward. The finding of a substantial ALDH^+^CD44^+^CD24^−^ (Figure 4A) population stands in contrast to our previous findings in cancer (Liu et al., 2013). ALDH^+^CD44^+^CD24^−^ cells had the highest mammosphere-forming potential. Interestingly, an analysis of the luminal portion of the human mammary gland identified that EpCAM^+^CD49f^+^ALDH^+^ cells were previously found to have no overlap with CD44^+^CD24^−^ (Shehata et al., 2012), while we identified clear overlaps between the ALDH^+^ and CD44^+^CD24^−^ population as well as a range of phenotypes within ALDH^+^ cells, illuminating the existence of ALDH^+^ populations outside of the luminal progenitor context (Eirew et al., 2012). ALDH1A3 was previously identified as the isoform with highest aldehyde dehydrogenase activity, by the Aldefluor assay (Marcato et al., 2011), which we corroborated with our RNA-seq data of the bulk ALDH^+^ population. However, at the single-cell level, we identified a small number of ALDH^+^ cells that expressed *ALDH1A1* and had mesenchymal phenotypes. Honeth et al. (2015) reported that ALDH1A1- and ALDH1A3-stained cells are exclusive from each other, with ALDH1A1 cells localizing in small lobules and ALDH1A3 cells localizing in the extralobular ducts. ALDH1A1 expression has also previously been shown to be important for mammosphere formation potential (Honeth et al., 2014), and normal breast tissues from women with BRCA1 mutations were enriched for ALDH1A1-positive cells (Honeth et al., 2015, Liu et al., 2008). Future efforts should focus on the development of methods to distinguish and isolate live human ALDH1A3 and ALDH1A1 cells for further characterization.

The EMT and the converse process, MET, are essential in the formation of cancer metastases. Emerging evidence points to EMT and MET not as static states but a continuum, with data showing that cells exhibit both phenotypes in development and cancer (Nieto, 2013). Induction of EMT in mammary epithelial cells by ectopic expression of the EMT transcription factors Twist or Snail generates CD44^+^CD24^−^ stem cells (Mani et al., 2008). Transient activation of TWIST1 in mammary epithelial cells can induce a stem-like phenotype, without full reversion to an epithelial state (Schmidt et al., 2015). Furthermore, certain populations of circulating tumor cells display a dual epithelial/mesenchymal phenotype (Aceto et al., 2014). Single-cell profiling of normal and cancerous breast cells showed that early circulating breast cancer cells have a phenotype that resembles that of a normal breast stem cell (Lawson et al., 2015).

We identified a subpopulation of ALDH^+^ breast cells that were highly enriched for “stemness” genes that also expressed a hybrid of epithelial and mesenchymal markers. Additionally, genes overexpressed in this subpopulation were overexpressed in TNBC and also predictive of survival in TNBC, suggesting that the pathways regulating this subpopulation of cells are important in TNBC. Others have also begun to report on the properties of epithelial/mesenchymal hybrid cells in the breast. Culture of murine mammary epithelial EpH4 cells transiently treated with transforming growth factor β1 led to the development of hybrid cells, with increased clonogenicity and a more invasive phenotype (Bidarra et al., 2016). Additionally, single-cell qPCR analysis of CD44^+^ cells isolated from mammospheres from oncogene-immortalized mammary epithelial cells identified an epithelial/mesenchymal phenotype gene signature (Grosse-Wilde et al., 2015). Mathematical modeling, validated with *in vitro* cultures, predicts that Notch-Jagged signaling regulates the emergence of hybrid epithelial/mesenchymal cells (Boareto et al., 2016). Intriguingly, a recent study found a subset of ALDH^+^/CD49f^+^/EpCAM^+^ cells in normal tissue adjacent to a breast cancer expressed a hybrid basal/luminal phenotype (Anjanappa et al., 2017). We found that breast epithelial/mesenchymal cells were also enriched Notch signaling pathway members. Further elucidation of the biological processes that regulate these epithelial/mesenchymal hybrid cells will likely provide new targets for the prevention and treatment of metastatic cancer.

This study highlights the advantages of assaying sorted cell populations and single cells, rather than bulk tissue, to provide new insights into mammary gland biology. From our RNA-seq analysis of ALDH^+^ cells, ALDH^+^ cells express genes associated with both epithelial and mesenchymal cell states. However, it was not until we quantified single-cell gene expression that we observed that there are subsets of epithelial-like, mesenchymal-like, and hybrid epithelial/mesenchymal cells. Understanding the factors that regulate the relative proportions of these cells is likely important for understanding the risk of developing different breast cancer subtypes. While our study here focused on reduction mammoplasty tissues for single-cell profiling, future studies should also assay breast punch biopsies from true “normal” volunteers, due to the findings that reduction mammoplasty tissues may not accurately reflect the true “normal” breast state (Degnim et al., 2012). Future work, taking advantage of new advances in single-cell transcriptomics, such as Drop-Seq (Macosko et al., 2015), will undoubtedly reveal additional levels of complexity in human mammary stem cell regulation. Even with a more limited spectrum of genes analyzed in a single breast stem cell transcriptional profile, we found striking inter- and intraindividual heterogeneity in mammary stem cell populations and genes and pathways that help define this heterogeneity. Further understanding of the intrinsic and extrinsic factors that affect breast stem cell heterogeneity will likely provide insights for the prevention, early detection, and treatment of breast cancer.

## Experimental Procedures

### Tissue Procurement

Non-pathogenic breast tissue was isolated from women undergoing voluntary reduction mammoplasty at the University of Michigan. The study protocol was approved by the University of Michigan Institutional Review Board. Breast tissue was mechanically and enzymatically digested as previously described (Dontu et al., 2003, Kakarala et al., 2010). For immunofluorescence imaging, histologically normal tissues adjacent to breast cancer were collected from women (n = 4) undergoing mastectomy at the Karmanos Cancer Institute.

### Flow Cytometry

Mammary cells were stained for CD44 and CD24 expression and ALDH activity as described previously (Colacino et al., 2016). In brief, single mammary cells were first incubated with a lineage-depletion cocktail that consisted of biotinylated antibodies targeted against CD45, HLA-DR, CD14, CD31, CD41, CD19, CD235a, CD56, CD3, CD16, and CD140b (all from eBioscience, except for CD140b [Biolegend] and CD41 [Acris]). Next, cells were stained with Alexa Fluor 750-tagged streptavidin, LIVE/DEAD Fixable Dead Cell Stain (Invitrogen), CD24 (Biolegend), CD44 (Becton Dickinson), and Aldefluor (STEMCELL Technology). Single-color and isotype controls were included for compensation and gating purposes. Aldefluor-positive gating was based on DEAB (negative) controls. Flow-cytometry data analysis was performed with FlowJo software version 10.0.8.

### RNA Extraction and Sequencing

ALDH^+^, CD44^+^, and CD24^+^ cell populations were collected from three independent donors and RNA was isolated using the RNEasy Micro Kit (Qiagen) with on-column DNase treatment. RNA concentration and quality was determined using a NanoDrop (Thermo Fisher) and Bioanalyzer (Agilent). Ribosmal RNAs were depleted using Ribominus (Life Technologies) and sequencing libraries prepared with the SMARTer Stranded RNA-Seq kit (Clontech). Libraries were multiplexed (4 per lane) and sequenced using paired-end 50 cycle reads on a HiSeq 2500 (Illumina) at the University of Michigan DNA Sequencing Core Facility.

### RNA-Seq Data Analysis

The Flux high-performance computer cluster at the University of Michigan was used for computational analysis. Sequencing read quality was assessed utilizing FastQC. Sequencing reads read in pair using SeqTK. The first three nucleotides of the first read were trimmed, as recommended by Clontech, using Prinseq 0.20.3. Read pairs were aligned to the genome using STAR (Dobin et al., 2013), using the options “outFilterMultimapNmax 10” and “sjdbScore 2.” Aligned reads were assigned to GRCh37 genes using HTSeq-count, with the set mode “union.” We conducted differential expression testing utilizing edgeR (Robinson et al., 2010). Separate comparisons were conducted for each cell type (ALDH^+^, CD44^+^, and CD24^+^), adjusting for study subject as a covariate using glmLRT. To reduce the dispersion due to lowly expressed genes, we excluded from analysis genes with a mean read count of less than 5 across all samples. Normalized counts per million were estimated utilizing the “cpm” function in edgeR (Robinson et al., 2010). Genes were considered differentially expressed between cell populations at an FDR adjusted p value of less than 0.05 (Benjamini and Hochberg, 1995).

### Pathway Analyses and Integration with Publicly Available Data

Differentially expressed pathways were identified utilizing iPathwayGuide (AdvaitaBio). Biological pathways were considered differentially expressed at an FDR p < 0.05. To compare how genes identified as differentially expressed between the ALDH^+^ and CD44^+^ cells overlap with previously reported breast stem cell gene expression signatures, we compared log fold changes in expression in our data for genes identified as uniquely upregulated (log_FC_ > 1) in CD49f^+^/EpCAM^−^ (“mammary stem”) and CD49f^+^/EpCAM^+^ (“luminal progenitor”) cell populations (Lim et al., 2009). Comparisons of the Cancer Genome Atlas expression data between TNBC and other cancers were conducted using Oncomine (https://www.oncomine.org/resource/login.html). Estimation of recurrence-free survival differences by expression of specific genes was conducted using KMPlot (Györffy and Schäfer, 2009) for four gene probes: 209086_x_at (*CD146*), 203237_s_at (*NOTCH3*), 213342_at (*YAP1*), and 214031_s_at (*KRT7*). Survival analyses were restricted to estrogen receptor-negative, progesterone receptor-negative, HER2-negative tumors (N = 249 patients in total), with groups dichotomized using the autoselected best cutoff. Survival based on the mean expression of these four probes was also estimated.

### Mammosphere Formation

Single cells were sorted from reduction mammoplasty from three individuals by FACS as described above and plated in 96-well ultralow attachment plates (Corning) at a density of 500 cells per well. Four populations were isolated: ALDH^+^CD44^+^CD24^−^, ALDH^−^CD44^+^CD24^−^, ALDH^+^ that are not CD44^+^CD24^−^, and ALDH^−^ that are not CD44^+^CD24^−^. Mammospheres formed for 7–10 days in Mammocult medium (STEMCELL). Each experiment was run with at least five technical replicates per condition, and the primary sphere number was quantified manually.

### Single-Cell Transcriptional Profiling

Single ALDH^+^CD44^+^CD24^−^ and ALDH^+^ that are not CD44^+^CD24 mammary epithelial cells sorted by flow cytometry (explained above) were stained with CellTracker dyes to differentiate the two populations and loaded onto the Fluidigm's C1 PreAmp chip to isolate single cells. C1 chips were examined under an Olympus IX83 fluorescent microscope to verify that chambers contained single cells. The captured cells then sequentially underwent lysis, RNA release, cDNA synthesis, and preamplification of 96 target genes. cDNAs were analyzed using Fluidigm's Biomark HD system, 96 × 96 chip, and 96 TaqMan assays to determine expression patterns of 96 target genes in each cell. qPCR data were analyzed using SINGuLAR to generate violin, heatmap clustering, and principal component analysis plots. Differential expression analyses between populations were conducted by t test or ANOVA of log_2_ expression values.

### Immunofluorescence Staining

Snap-frozen tissue sections obtained from the Karmanos Cancer Institute were transferred from storage at −80°C to a −20°C freezer 15 min prior to processing. Sections were fixed with 4% paraformaldehyde (Electron Microscopy Sciences) at 4°C for 15 min and then washed with PBS (pH 7.4) (Thermo Fisher Scientific). Fixed sections were then permeabilized with an ice-cold solution of 1:1 methanol/acetone for 1 min. To reduce non-specific adhesion, we incubated sections for 1 hr with a blocking buffer composed of 5% goat serum (Sigma-Aldrich) diluted in PBS. Primary antibodies targeting ALDH1A3 (4.3 μg/mL, LSBio: LS-C172937), Cytokeratins 8 + 18 (1:200 dilution, Abcam: ab194130), and vimentin (5 μg/mL, Thermo Fisher Scientific: MA1-10459) were diluted in blocking buffer and applied overnight in a humidified chamber at 4°C. Sections were washed three times for 5 min each with PBS. The following directly conjugated and secondary antibodies were diluted in blocking buffer and applied for 6–8 hr in a humidified chamber at 4°C: ALDH1A1 AF647 (10 μg/mL, Abcam: ab195255), CD44 BV510 (2 μg/mL, BioLegend: 103043), goat anti-guinea pig immunoglobulin G (IgG) DL755 (2.5 μg/mL, Thermo Fisher Scientific: SA5-10099), goat anti-mouse IgG1 AF488 (2 μg/mL, Thermo Fisher Scientific: A-21121), and rat anti-mouse IgM phycoerythrin (2 μg/mL, BioLegend: 406507). Sections were again washed three times for 5 min each with PBS, then treated with DAPI (1 μg/mL, Thermo Fisher Scientific) to label nuclei. Finally, the tissue sections were mounted with coverslips using Prolong Diamond Antifade Mountant (Thermo Fisher Scientific) and imaged with an Olympus IX-83 microscope using up to six optical filter cubes corresponding to each fluorophore.

## Author Contributions

J.A.C., E.A., L.S.R., and M.S.W. conceived of the study. J.A.C., E.A., M.D.B., R.H., and S.F. conducted the experiments. J.M., J.B., and M.L.C. collected patient samples. J.A.C., E.A., M.D.B., R.H., S.P.M., M.L., D.H., M.A.S., L.S.R., and M.S.W. analyzed the data. J.A.C., E.A., and M.S.W. wrote the manuscript. All authors edited the manuscript. All authors approved the final version.

## Accession Numbers

RNA-seq fastq files are publically available for download (ArrayExpress: E-MTAB-5585).

## Supplemental Information

Document S1. Figures S1–S6

Table S1. Comparison of Expression of All Genes between ALDH^+^ and ALDH^−^CD44^−^CD24^+^ Cells

Table S2. Most Enriched KEGG Pathways for Genes Differentially Expressed between ALDH^+^ and ALDH^−^CD44^−^CD24^+^ Cells

Table S3. Comparison of Expression of All Genes between ALDH^+^ and ALDH^−^CD44^−^CD24^+^ Cells

Table S4. Comparison of Gene Expression between Samples of Bulk RNA, Normalized for Housekeeping Genes, Isolated from ALDH^+^ Cells that Expressed CD44^+^/CD24^−^ (Dual) and Did Not Express CD44^+^/CD24^−^ (ALDH^+^)

Table S5. Comparison of Single-Cell Gene Expression Levels between the Four Identified Expression Clusters of ALDH^+^ Normal Mammary Cells

Table S6. Comparison of Single-Cell Gene Expression Levels between ALDH^+^ Normal Mammary Cells Expressing Detectable Levels of *ALDH1A1* (n = 9) Compared with Cells that Did Not (n = 96)

Document S2. Article plus Supplemental Information
