# Supplementary material for: Heterogeneity of Human Breast Stem and Progenitor Cells as Revealed by Transcriptional Profiling
Source: Stem Cell Reports. 2018 Mar 29;10(5):1596–609. doi: 10.1016/j.stemcr.2018.03.001 (PMC5995162; doi:10.1016/j.stemcr.2018.03.001)

Stem Cell Reports, Volume *10*

Supplemental Information

Heterogeneity of Human Breast Stem and Progenitor Cells as Revealed by Transcriptional Profiling

Justin A. Colacino, Ebrahim Azizi, Michael D. Brooks, Ramdane Harouaka, Shamileh Fouladdel, Sean P. McDermott, Michael Lee, David Hill, Julie Madden, Julie Boerner, Michele L. Cote, Maureen A. Sartor, Laura S. Rozek, and Max S. Wicha

Supplemental Table Legends

Supplemental Table 1. Comparison of expression of all genes between ALDH^{+} and ALDH^{-}CD44^{-}CD24^{+} cells.

Supplemental Table 2. Most enriched KEGG pathways for genes differentially expressed between ALDH^{+} and ALDH^{-}CD44^{-}CD24^{+} cells.

Supplemental Table 3. Comparison of expression of all genes between ALDH^{+} and ALDH^{-}CD44^{-}CD24^{+} cells.

Supplemental Table 4. Comparison of gene expression between samples of bulk RNA, normalized for housekeeping genes, isolated from ALDH+ cells that expressed CD44+/CD24- (Dual) and did not express CD44+/CD24- (ALDH+).

Supplemental Table 5. Comparison of single cell gene expression levels between the 4 identified expression clusters of ALDH+ normal mammary cells.

Supplemental Table 6. Comparison of single cell gene expression levels between ALDH+ normal mammary cells expressing detectable levels of *ALDH1A1* (n=9) compared to cells that did not (n=96).

Supplemental Figure 1. Differential expression of genes in the (A) Ribosome KEGG pathway, (B) the Proteosome KEGG pathway, and (C) the Oxidative Phosphorylation KEGG pathway between ALDH^{+} and ALDH^{-}CD44^{-}CD24^{+} cells.

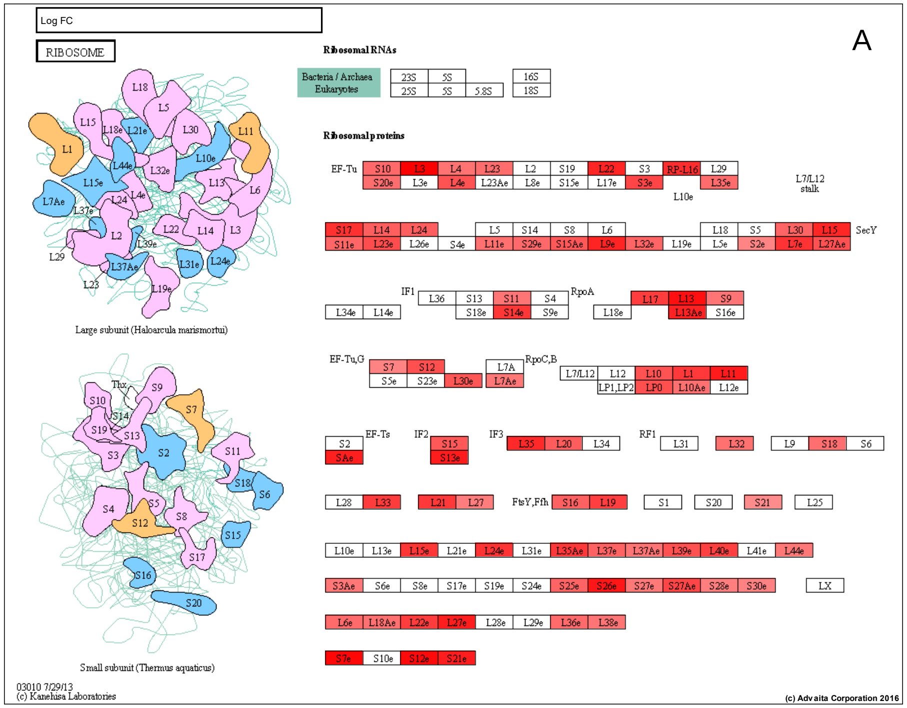

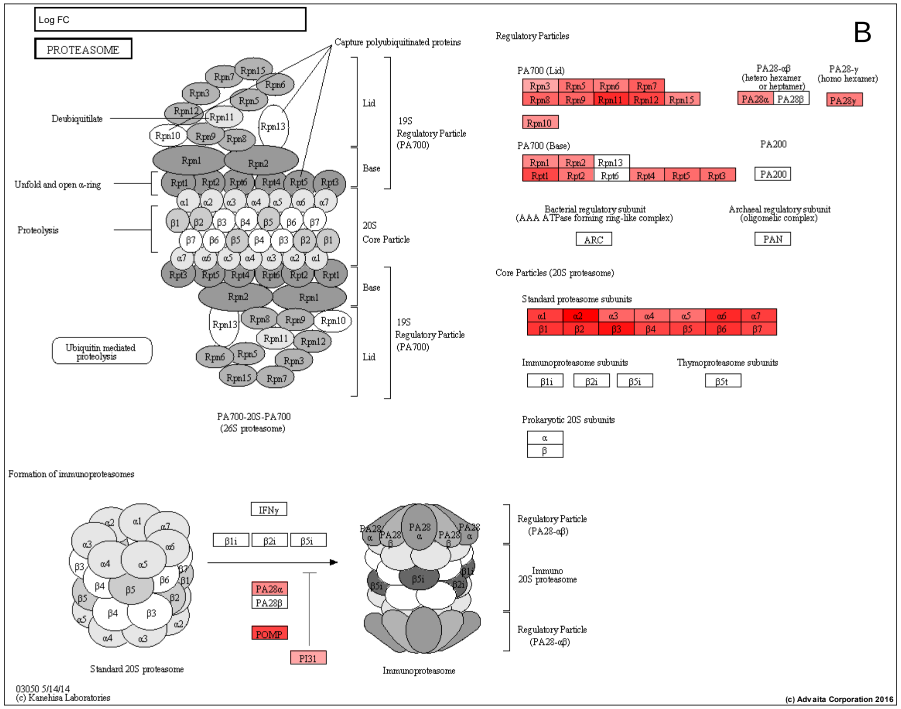

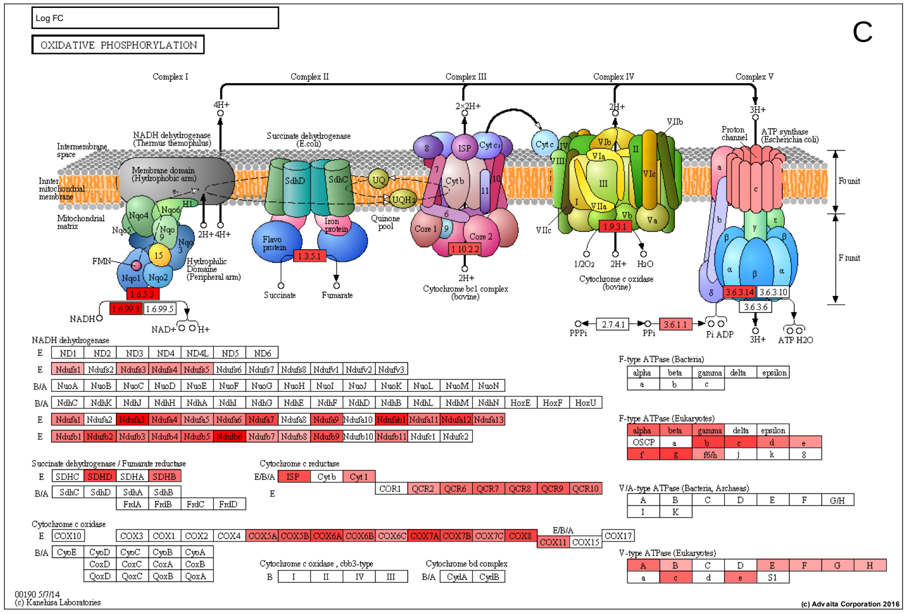

Supplemental Figure 2. Differential expression of genes in the (A) Proteosome KEGG pathway, (B) ECM-Receptor Interactions KEGG pathway, (C) Focal Adhesion KEGG pathway, and (D) PI3K-AKT signaling KEGG pathway between $ALDH^{-}CD44^{+}CD24^{-}$ and $ALDH^{-}CD44^{-}CD24^{+}$ cells.

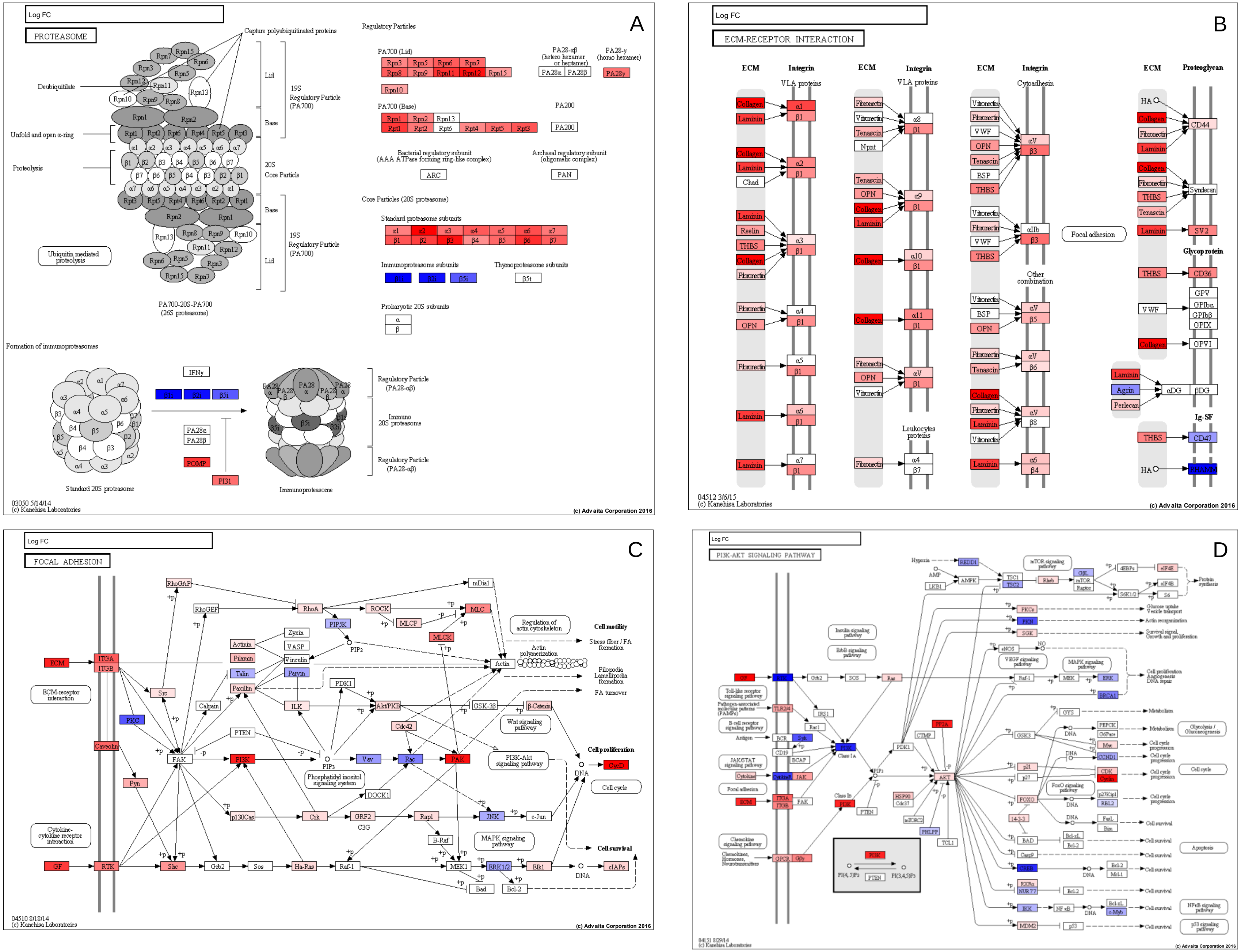

Supplemental Figure 3. Profiling of normal human breast cells using a custom 96 gene panel on the Fluidigm Biomark and C1 instruments. (A) The RNA-seq data of the 96 genes on the custom panel is sufficient to clearly distinguish normal mammary cell populations. (B) Evaluation of bulk RNA, normalized for housekeeping genes, isolated from ALDH+ cells that expressed CD44+/CD24- (Dual) and did not express CD44+/CD24- (ALDH+).

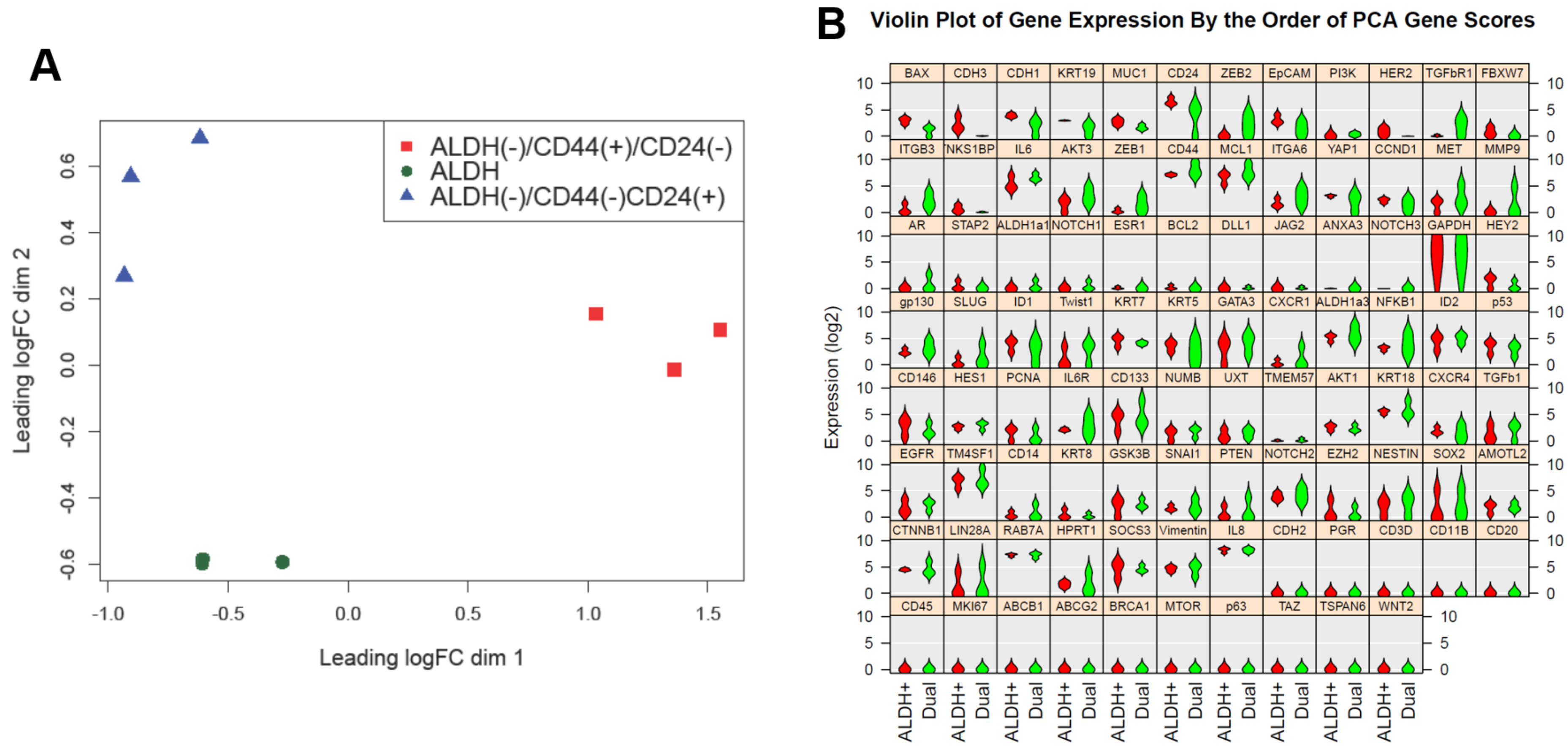

Supplemental Figure 4. Distribution of individual cells across the four single cell expression clusters by (A) individual from which the cells were collected or (B) Expression of $ALDH^{+}$ Bulk ($ALDH^{+}$ cells which do not express $CD44^{+}CD24^{-}$) or $ALDH^{+}CD44^{+}CD24^{-}$ quantified by flow cytometry.

A

	NM11	NM15	NM17	Total
Cluster 1	3	0	3	6
Cluster 2	3	21	4	28
Cluster 3	10	0	3	13
Cluster 4	20	18	20	58
Total	36	39	30	105

Chi-Sq Test Stat = 34.1, p=6.3E-6

B

	ALDH Bulk	Dual	Total
Cluster 1	3	3	6
Cluster 2	17	11	28
Cluster 3	4	9	13
Cluster 4	38	20	58
Total	62	43	105

Chi-Sq Test Stat = 5.5, p=0.13

Supplemental Figure 5. Immunofluorescence antibody staining of adjacent normal breast tissue for DAPI, ALDH1A1, ALDH1A3, CK8/18, and Vimentin, and for panel (C), also CD44. (A) Scale bar = 100µm (B) Scale bar = 10µm, arrow identifies an ALDH1A3^{+}, Vimentin^{+} cell (C) Yellow arrow identifies a CD44^{+},Vimentin^{+}, ALDH1A1^{+} cell, while white arrow identifies an ALDH1A3^{+}, CD44^{+}, CK8/18^{+} cell.

Arrow identifies cell with co-expression of CK8/18, Vimentin, and ALDH1A3. Scale bar = 10µm.

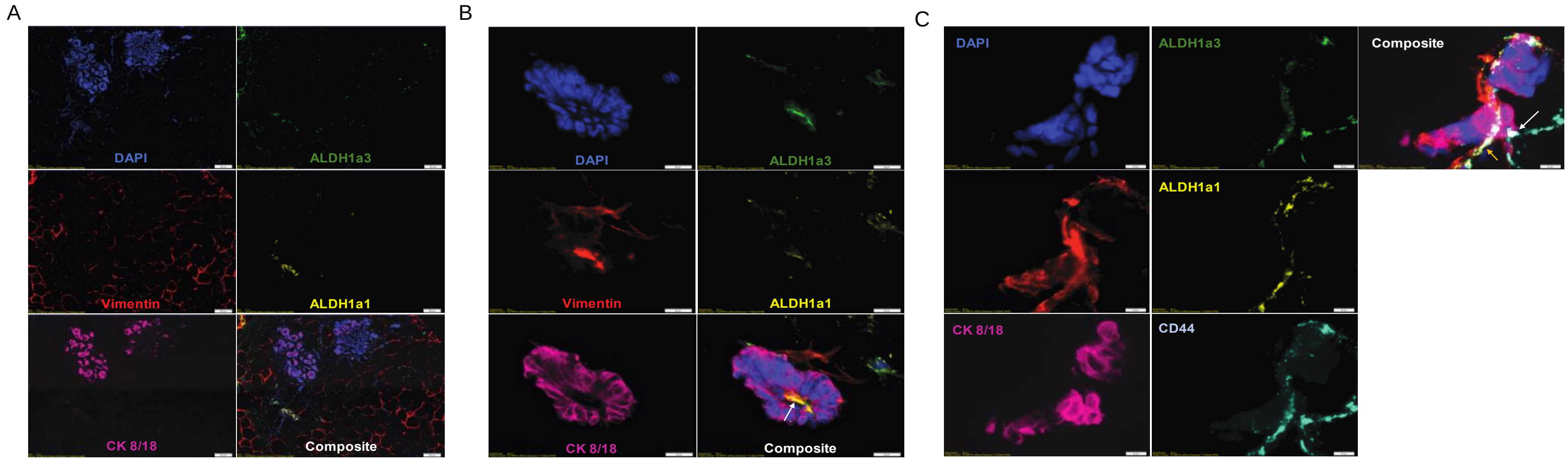

Supplemental Figure 6. Violin plot, with genes ordered by statistical significance, comparing gene expression between normal mammary cells expressing detectable levels of *ALDH1A1* (n=9) compared to cells that did not (n=96).

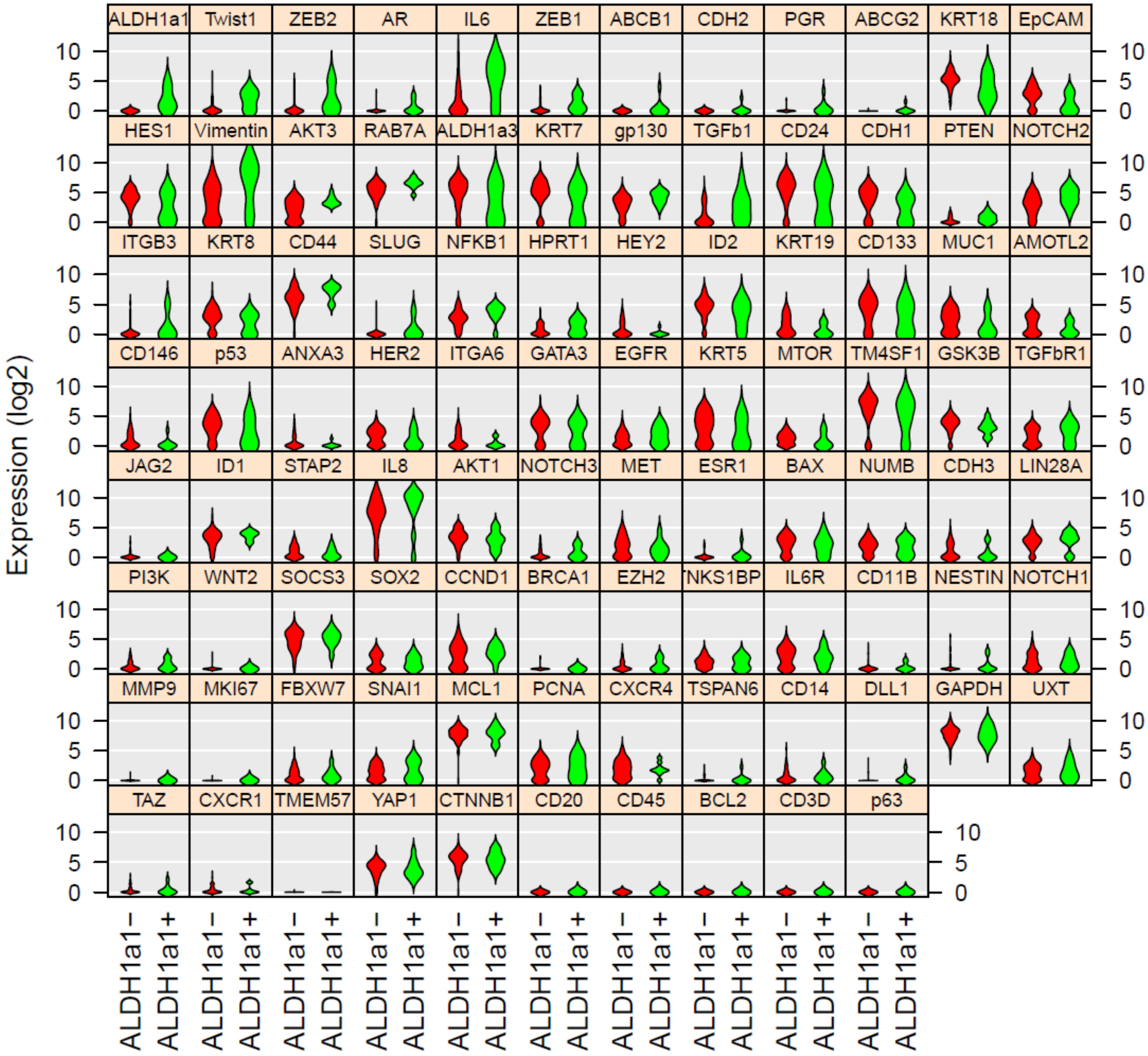

Supplement: Document S1. Figures S1–S6 [file mmc1.pdf]
